# Fall and Winter Temperatures, Together with Spring Temperatures, Determine the First Flowering Date of *Prunus armeniaca* L.

**DOI:** 10.3390/plants14101503

**Published:** 2025-05-16

**Authors:** Di Tang, Brady K. Quinn, Yunfeng Yang, Liang Guo, David A. Ratkowsky, Peijian Shi

**Affiliations:** 1 Co-Innovation Center for Sustainable Forestry in Southern China, College of Landscape Architecture, Nanjing Forestry University, 159 Longpan Road, Nanjing 210037, China; dtang@njfu.edu.cn; 2 St. Andrews Biological Station, Fisheries and Oceans Canada, 125 Marine Science Drive, St. Andrews, NB E5B 0E4, Canada; brady.quinn@dfo-mpo.gc.ca; 3 Institute of Soil and Water Conservation, Northwest A & F University, 26 Xinong Road, Xianyang 712100, China; guoliang2014@nwsuaf.edu.cn; 4 Tasmanian Institute of Agriculture, University of Tasmania, Private Bag 98, Hobart, TAS 7001, Australia; 5 Co-Innovation Center for Sustainable Forestry in Southern China, Bamboo Research Institute, Nanjing Forestry University, 159 Longpan Road, Nanjing 210037, China

**Keywords:** apricot, Arrhenius equation, generalized additive model, linear equation, temperature-dependent developmental rate

## Abstract

Chilling and spring temperature accumulation are both considered key factors determining the timing of the spring bloom in many flowering plants. The accumulated developmental progress (ADP) method predicted the first flowering date (FFD) of a species of Rosaceae well in a previous study. However, whether this approach can be applied to other species, and whether the prediction errors in FFD based on the ADP method can be further accounted for by fall and winter temperatures (FWTs), remains unknown. The ADP method and two others were tested using a 39-year apricot FFD data series. The goodness of fit obtained with each method was assessed using the root mean square error (RMSE) between the observed and predicted FFDs. We used the residuals obtained using the ADP method as a response variable to fit generalized additive models (GAMs) including six FWTs as predictors. The GAMs generated based on different combinations of predictors were compared using Akaike’s information criterion (AIC) to test whether using FWTs can reduce prediction error. The ADP method had the lowest RMSE, which equaled 3.0904 days. Together, the number of cold days, the number of chilling hours, the mean value of the daily maximum temperatures, and the mean value of the daily mean temperatures from 1 November of the preceding year to the starting date accounted for 96% of the deviance in the residuals obtained using the ADP method. Including these predictors reduced the RMSE to 0.6162 days. The ADP method is a valid technique to quantify the effect of spring temperatures from a given starting date on the FFD. The FWTs and the number of cold days can also influence the FFD. The present work provides evidence that FWTs including daily maximum temperatures and spring mean temperatures together determine the FFD of apricot.

## 1. Introduction

Phenology is closely associated with the services and functioning of ecosystems involved in agriculture, fishery, forestry, and horticulture [1,2]. Global climate warming has strongly impacted the timing of many phenological events of animals and plants [3,4,5]. Such shifts can lead to a mismatch between the phenological occurrence of plants and that of herbivores, as well as a subsequent mismatch between herbivores and carnivores [6]. Spring phenology is always a focus of phenological investigation because the growth and development of many poikilotherms and plants are temperature-dependent, and such organisms usually break dormancy and diapause in response to gradually increasing daily air temperatures in this season in the temperate zone of the Northern Hemisphere [3]. The currently popular viewpoint is that the timing of spring phenology is determined both by fall and winter low temperatures and spring mean temperatures [4,7]. The spring phenology of plants can be advanced by increasing spring temperatures but delayed by increasing fall and winter temperatures due to the reduction in fall and winter chilling accumulation [4]. The extent of fall and winter chilling accumulation has always been considered to have a significant influence on the spring phenology of plants, such as the timing of bud sprouting, flowering, and leaf unfolding, and several models of these relationships have been proposed [8,9]. Relative to the influence of spring temperatures, the influence of fall and winter temperatures (FWTs) on the occurrence time of spring phenological events has seldom been tested, with the exception of a few studies [10,11,12,13]. Most prior studies hypothesized that FWTs make a smaller contribution to the occurrence time of spring phenology than that of spring temperatures and that the relative contributions of these predictors are species-specific [10]. However, whether this hypothesis holds true generally remains unknown.

The spring phenology of plants, especially the timing of key developmental events in early spring, is related to the breaking of two stages of dormancy: endo-dormancy that can only be broken by a certain chilling accumulation and eco-dormancy that can only be broken by a certain heat accumulation [14,15]. Chilling accumulation is considered to play a critical role in breaking endo-dormancy, and after the completion of endo-dormancy, the accumulation of spring effective temperatures is considered to play a critical role in breaking eco-dormancy [14,16,17]. Significant interspecific variation in chilling requirements (CRs) and heat requirements (HRs) has been documented in apricot (*Prunus armeniaca* L.) [18]. Studies quantified CRs across cultivars as ranging from 596 to 1266 chilling units, revealing a negative correlation between CRs and HRs. This variability underscores the necessity of cultivar-specific agroclimatic assessments, particularly under climate change scenarios where incomplete CRs fulfillment may lead to erratic flowering and yield instability [19]. For the latter process (i.e., the breaking of eco-dormancy), the effect of spring temperatures on the time of occurrence can be clearly explained by experimental evidence from thermal biology in arthropods and plants ([20,21,22] and references therein). The effect of temperature (*T*) on the developmental rate (denoted as *r*, i.e., the reciprocal of the developmental duration for completing a specific developmental stage, which represents the developmental progression per unit time) is a left-skewed bell-shaped curve. At low temperatures, *r* exponentially increases with *T*; at moderate temperatures, *r* increases approximately linearly with *T*; at high temperatures, *r* steeply decreases with *T* [23,24,25]. There are many mathematical models available that describe the temperature-dependent developmental rate of arthropods and plants [20,21,26,27]. In early spring, daily mean temperature (denoted as *T*_mean_) is usually lower than 20 °C, so the temperature-dependent development rate can be approximated by a linear or exponential equation [23,24,25,28]. Because the developmental rate does not decrease in low and mid-temperature ranges, some investigators even use the logistic equation to describe the effect of temperature on developmental rate [29] and then apply this approach to account for the effect of spring temperatures (referred to as forcing requirement in many studies on phenology) on the breaking of eco-dormancy [30]. Regardless of whether the developmental rate–temperature relationship is thought of as a straight line or a curve, the timing of a particular phenological event can be predicted by calculating the cumulative developmental rates associated with the daily *T*_mean_ values in a period from a starting date to the occurrence time, adding up to 100% [11,12,31]. Using some optimization methods to minimize the root mean square error (RMSE) between the observed and predicted occurrence dates, the starting date (in day-of-year) and the parameters of the developmental rate equation can then be determined. This approach is referred to as the accumulated developmental progress (ADP) method [11,12]. The ADP method was found to be better than the traditional accumulated degree-day (ADD) method. However, prior studies have usually reported large prediction errors for both the ADD and ADP methods, reflected by their RMSE ranging between 2 and 5 days between the observed and predicted occurrence times [10,11,12,32,33]. Although some mathematical models have already been proposed to describe the effect of fall and winter low temperatures on the break of endo-dormancy, these methods are usually based on species-specific experiments, and the model parameters lack generalizability to other species [13,14,16,30]. In addition, these models usually require that the parameters of auxiliary models are predetermined, which introduces a certain degree of subjectivity into their determination [13]. It should also be noted that the prediction errors resulting from such models are large, probably because of small sample size (i.e., the number of years for which there are phenological records) or probable problems inherent in these methods themselves.

The main reason that temperature-dependent developmental rate models have large prediction errors is that those models neglect the effect of FWTs on the break of endo-dormancy; further, the main reason that the existing models that consider the effects of fall and winter temperatures and spring temperatures on spring phenology also have large prediction errors is that the mechanisms by which FWTs impact phenology have been not as clearly elucidated as those of spring temperatures have so far [13]. Many models of the effect of FWTs on the break of endo-dormancy lack sufficiently convincing experimental evidence to be used as a general tool [13,14,16,30]. In this context, Shi et al. [11] proposed a method to solve this problem based on the first flowering date (FFD) time series of a species of Rosaceae, the cherry *Prunus × yedoensis* Matsum. First, the ADP method was used to calculate the predicted occurrence dates (in day-of-year). Second, the residuals calculated between the observed and predicted occurrence dates were further analyzed using three fall and winter low temperature measures: (i) the number of cold days with daily minimum temperature (*T*_min_) ≤ −5.6 °C; (ii) the mean of the *T*_min_ values from 1 November of the preceding year to the starting date for the ADP method; and (iii) the minimum annual temperature. Using the above protocols, the RMSE value for predicting the FFD of *P. yedoensis* decreased from 4.29 to 2.80 days. However, two issues were not addressed by this approach. One is whether the protocols proposed by ref. [11] apply to the FFD of other species, and another is whether the prediction error can be further decreased by introducing other measures of FWTs into predictive models; e.g., the daily maximum temperatures (*T*_max_) in fall and winter that have been largely neglected in prior work. For instance, increased fall and winter *T*_max_ likely leads plants to finish accumulating effective temperatures earlier, which might lead to spring phenological events happening sooner in the year.

In the present study, we used a 39-year FFD time series of *P. armeniaca* at a representative site to test the validity of the ADP method and examine whether FWTs, including the daily minimum, maximum, and mean temperatures in fall and winter, as well as the number of cold days with *T*_min_ less than a certain low temperature in the fall and winter seasons, can significantly influence the prediction of FFD in this species of Rosaceae.

## 2. Materials and Methods

### 2.1. First Flowering Date Records and Climate Data

First flowering date (FFD) data for *P. armeniaca* came from the Chinese Phenological Observation Network, which were recorded at the Summer Palace of Beijing, China (39°54′38″ N, 116°8′28″ E, 50 m above sea level) from 1963 to 2010, with the exceptions of 1969–1971 and 1997–2002 [34]. In total, there are 39 years of FFD records in the data set. Daily climate data of Beijing from 1962 to 2010 came from the China Meteorological Data Service Centre (https://data.cma.cn/en; accessed on 4 April 2014). The FFD and climate data are included in the R package “spphpr” (v1.1.4) [35].

### 2.2. Methods for Quantifying the Effect of Spring Temperatures on Phenological Occurrence Date

#### 2.2.1. Accumulated Degree Days (ADD) Method

This method is based on the hypothesis that the development rate, *r*, increases approximately linearly with temperature, *T*, which is expressed as a linear equation:

(1) r=a+bT,

where *a* and *b* are constants to be estimated. The temperature associated with *r* = 0 is defined as the lower developmental threshold temperature (or the “biological zero degree”), denoted as *T*_0_, where $T_{0}=-a/b$. When *T* < *T*_0_, *r* is considered to be zero. Equation (1) can alternatively be written as follows:

(2) $$k=D\left(T-T_{0}\right),$$

where *k* is the accumulation of effective temperatures (i.e., temperatures greater than the biological zero degree) required for completing a particular developmental stage, and *D* is the developmental duration, i.e., the reciprocal of *r*. In this representation, it is apparent that *k* = 1/*b*. It must be noted that *r* and *k* in Equations (1) and (2) are calculated as functions of constant experimental temperature. However, daily air temperatures in nature are variable. To capture this, Equation (2) can thus be rewritten as follows:

(3) $$k_{i}=\sum_{j=S}^{E_{i}}\left(T_{ij}-T_{0}\right),$$

where the subscript *i* represents *i*th year, *S* represents the starting date (in day-of-year) when the accumulation of effective temperatures starts, *E_i_* represents the actual occurrence date of a particular phenological event, the subscript *j* represents the *j*th day (day-of-year), and *T_ij_* represents the daily mean temperature of the *j*th day of the *i*th year. If one assumes that there are *n*-year phenological records, then there is a series of *k_i_* values, i.e., *k*_1_, *k*_2_, *k*_3_, …, *k_n_*.

In theory, the accumulation of effective temperatures required to complete development to a particular stage or phenological event’s occurrence is a constant value across different years. However, the *k_i_* values calculated from empirical observations show some variation across different years. The mean of the *k_i_* values, denoted as $\bar{k}$, is regarded as the expected required accumulation of effective temperatures. Here, *S* and *T*_0_ are unknown parameters that need to be estimated. The following protocols for determining the two parameters were recommended by Aono [33]. First, the mean value of the daily mean temperatures between the starting date of observations and the observed occurrence date is hypothesized to be negatively correlated with the observed occurrence date. This means that a higher mean temperature during the developmental period can result in the phenological event occurring at an earlier date. A group of candidate *S* values are provided, and the value associated with the lowest correlation coefficient between the mean of the daily mean temperatures from the starting date and the observed occurrence date is determined as the estimated value of *S*. Second, a group of candidate *T*_0_ values are provided. Using each candidate *T*_0_ value, the predicted occurrence date in the *i*th year is determined by the following steps. When $\sum_{j=S}^{F_{i}}\left(T_{ij}-T_{0}\right)=\bar{k}$ (where *F_i_* > *S*), *F_i_* is the predicted occurrence date in the *i*th year; when $\sum_{j=S}^{F_{i}}\left(T_{ij}-T_{0}\right)<\bar{k}$ and $\sum_{j=S}^{F_{i}+1}\left(T_{ij}-T_{0}\right)>\bar{k}$, the trapezoid method [32,36] is used to determine the predicted occurrence date in the *i*th year. The root mean square error (RMSE) calculated between the observed and predicted occurrence dates is then used to determine the numerical value of *T*_0_, such that the candidate *T*_0_ value associated with the lowest RMSE is selected as the estimated value of *T*_0_.

#### 2.2.2. Accumulated Days Transferred to a Standardized Temperature (ADTS) Method

This method is based on the hypothesis that *r* increases approximately exponentially as *T* (in K) increases, which can be expressed using the Arrhenius equation [11,33,37]:

(4) $$r=A\;\exp\left(-\frac{E_{a}}{RT}\right)=\exp\left(B-\frac{E_{a}}{RT}\right)$$

where *A* is a pre-exponential constant to be estimated, *B* = log(*A*), *E_a_* is the activation free energy (in kcal∙mod^−1^) of a biochemical reaction controlling developmental progression, and *R* is the universal gas constant (=1.987 × 10^−3^ kal∙mol^−1^∙K^−1^). In practice, to reduce the value of *B* during parameter estimation, the pre-exponential constant is multiplied by 10^12^, i.e., $r=10^{12}\;\exp\left(B-E_{a}/RT\right)$.

According to the definition of *r*, it represents the developmental progress per unit time (e.g., per day) and is the reciprocal of the developmental duration *D*, i.e., *r* = 1/*D*. Let *T_s_* represent a standard temperature (in K, e.g., 25 °C = 298.15 K) and *r_s_* represent the developmental rate at this standard temperature. Let *r_j_* represent the developmental rate at *T_j_*, an arbitrary absolute temperature (in K). Given that $D_{s}r_{s}=D_{j}r_{j}=1$, it follows that

(5) $$\frac{D_{s}}{D_{j}}=\frac{r_{j}}{r_{s}}=\exp\left[\frac{E_{a}\left(T_{j}-T_{s}\right)}{RT_{j}T_{s}}\right],$$

where $D_{s}/D_{r}$ is termed the number of days transferred to a standardized temperature (DTS) [33,37]. If one replaces *T_j_* in Equation (5) with *T_ij_* (i.e., the daily mean temperature of the *j*th day of the *i*th year), then the annual accumulated DTS of the *i*th year (denoted as *U_i_*) equals

(6) $$U_{i}=\sum_{j=S}^{E_{i}}\exp\left[\frac{E_{a}\left(T_{ij}-T_{s}\right)}{RT_{ij}T_{s}}\right].$$

As for the accumulation of effective temperatures described above, in theory, the annual accumulated DTS is a constant value, but calculated *U_i_* values exhibit some variation across different years. The mean of the *U_i_* values, denoted as $\bar{U}$, is regarded as the expected value. Here, *S* and *E_a_* are unknown parameters that need to be estimated. The following protocols to determine the two parameters have been recommended in previous studies [33,37]. First, different combinations of the candidate *S* values and candidate *E_a_* values are provided. Using each combination, the predicted occurrence date in the *i*th year is then determined by the following steps. When $\sum_{j=S}^{F_{i}}\exp\left[\frac{E_{a}\left(T_{ij}-T_{s}\right)}{RT_{ij}T_{s}}\right]=\bar{U}$ (where *F_i_* > *S*), *F_i_* is the predicted occurrence date in the *i*th year; when $\sum_{j=S}^{F_{i}}\exp\left[\frac{E_{a}\left(T_{ij}-T_{s}\right)}{RT_{ij}T_{s}}\right]<\bar{U}$ and $\sum_{j=S}^{F_{i}+1}\exp\left[\frac{E_{a}\left(T_{ij}-T_{s}\right)}{RT_{ij}T_{s}}\right]>\bar{U}$, the trapezoid method [32,36] is used to determine the predicted occurrence date in the *i*th year. The RMSE calculated between the observed and predicted occurrence dates is then used to determine the numerical values of *S* and *E_a_*. Specifically, the combination of the candidate *S* and *E_a_* values that is associated with the lowest RMSE is selected as that with the estimated values of *S* and *E_a_*. It should be noted that the ADTS method only applies when the temperature-dependent developmental rate equation is expressed as the Arrhenius equation.

#### 2.2.3. Accumulated Developmental Progress (ADP) Method

This method permits the user to predict the occurrence date using any defined temperature-dependent developmental rate equation, denoted as $r\left(P;T\right)$, where **P** represents the vector of the model parameter(s). In this method, when the annual accumulated developmental progress (i.e., the accumulated developmental rates) reaches 100%, the phenological event is predicted to occur for each year [11,12,31,38]. Let *V_i_* represent the annual accumulated developmental progress in the *i*th year, which equals

(7) $$V_{i}=\sum_{j=S}^{E_{i}}r_{ij}\left(P;\;T_{ij}\right).$$

In theory, all *V_i_* values are a constant value of 100%, although actual calculated *V_i_* values do vary somewhat across different years. The following approach is used to determine the predicted occurrence date. When $\sum_{j=S}^{F_{i}}r_{ij}=100\%$ (where *F_i_* > *S*), *F_i_* is the predicted occurrence date in the *i*th year; when $\sum_{j=S}^{F_{i}}r_{ij}<100\%$ and $\sum_{j=S}^{F_{i}+1}r_{ij}>100\%$, the trapezoid method [32,36] is used to determine the predicted occurrence date in the *i*th year. When the starting date (*S*) and $r\left(P;T\right)$ are known, the model parameters can be estimated using the Nelder–Mead optimization method [39] to minimize the RMSE between the observed and predicted occurrence dates, i.e.,

(8) $$\hat{P}=\arg\underset{P}{\min}\left\{\mathrm{RMSE}\right\}=\arg\underset{P}{\min}\sqrt{\frac{\sum_{i=1}^{n}{\left(E_{i}-{\hat{E}}_{i}\right)}^{2}}{n}},$$

where ${\hat{E}}_{i}$ represents the predicted occurrence date in the *i*th year. Because *S* is not determined in the model fitting here, a group of candidate *S* values (in day-of-year) need to be provided. Assuming that there are *m* candidate *S* values, i.e., *S*_1_, *S*_2_, *S*_3_, ∙∙∙, *S_m_*, then for each *S_q_* (where *q* ranges between 1 and *m*), a vector of the estimated model parameters, ${\hat{P}}_{q}$, can be generated by minimizing RMSE*_q_* using the Nelder–Mead optimization method [39]. Then, the $\hat{P}$ associated with min{RMSE_1_, RMSE_2_, RMSE_3_, ∙∙∙, RMSE*_m_*} is finally the target parameter vector.

### 2.3. Methods for Quantifying the Effect of Fall and Winter Temperatures (FWTs) on Occurrence Date

As described above, the ADD, ADTS, and ADP methods were used to predict the first flowering dates of apricots, and the method associated with the lowest RMSE between the observed and predicted occurrence dates was selected for use in subsequent analyses. The residuals between the observed and predicted occurrence dates were calculated and then treated as the response variable, *y*, in analyses of the effects of fall and winter temperatures (FWTs) on occurrence date prediction. The present study tested the following six FWT measures as potential predictors: the number of days with daily minimum temperature ≤ a critical low temperature (*x*_1_), the number of chilling hours (*x*_2_), the mean value of the daily minimum temperatures (*x*_3_), the mean value of the daily maximum temperatures (*x*_4_), the mean value of the daily mean temperatures (*x*_5_), and the minimum value of the daily minimum temperatures (i.e., the minimum annual temperature, *x*_6_); all these predictors were calculated from 1 November of the preceding year to the starting date. Generalized additive models (GAMs) [40,41,42] with different candidate combinations of the above predictors were generated and compared to examine the relationships of *y* to the above predictors (Table 1).

A GAM is a non-parametric fitting method for multiple predictors, and does not require that the influence of each predictor on the response variable is known in advance. Using the partial residual plots generated during GAM fitting [40,41,42], the influence of each predictor on the response variable can be readily observed. When the mechanisms by which the various FWT measures influence the occurrence date are not known, it is feasible to use GAMs to explore the forms (linear or non-linear performance) of the relationships between those predictors and the occurrence date. We selected the best model among the candidate GAMs that we tested as the one that had the highest percentage of deviance explained (%), provided that all smooth terms were significant at the 0.05 level. In addition, Akaike’s information criterion (AIC) [43,44] was used to compare models. The model with the lowest AIC was selected as the best, as the AIC value calculated for a model reflects the trade-off between its goodness of fit (decreases AIC) and model structural complexity (increases AIC).

The critical low temperature used to calculate the observed *x*_1_ was determined by selecting the critical low temperature associated with the largest percentage of deviance explained among a set of candidate critical low temperatures. The accumulated chilling hours (ACH) in the *i*th year (observed *x*_2_) were calculated using the Chilling Hours model [8,45,46]:

(9) $${\mathrm{ACH}}_{i}=\sum_{j=305\;\left(\mathrm{or}\;306\right)}^{365\;\left(\mathrm{or}\;366\right)}\sum_{w=1}^{24}1_{i-1,j,w}+\sum_{j=1}^{S-1}\sum_{w=1}^{24}1_{i,j,w},$$

where the first term on the right-hand side of Equation (9) represents the ACH from 1 November to 31 December of the preceding year, and the second term on the right-hand side of Equation (9) represents the ACH from 1 January to the starting date. The term $1_{i,j,w}$ is the indicator function, which equals

(10) $$1_{i,j,w}=\left\{\begin{array}{cc} 1, & \mathrm{if}\;0\;{}^{\circ}C<T_{i,j,w}<7.2\;{}^{\circ}C;\\ 0, & \mathrm{if}\;T_{i,j,w}\leq 0\;{}^{\circ}C\;\mathrm{or}\;T_{i,j,w}\geq 7.2\;{}^{\circ}C. \end{array}\right.$$

Here, $T_{i,j,w}$ is the temperature of the *w*th hour of the *j*th day of the *i*th year, which was calculated using the following formula [47]:

(11) $$T_{w}=\frac{T_{\max}-T_{\min}}{2}\times \sin\left(\frac{\pi }{12}\times w-\frac{\pi }{2}\right)+\frac{T_{\min}+T_{\max}}{2},$$

where *T*_min_ and *T*_max_ represent the daily minimum and maximum temperatures, respectively, and *w* ranges from 1 to 24.

To quantify the contribution rate (CR) of each predictor variable to the deviance explained by fitting a GAM with *z* variables (*z* ≤ 6) to the data, the following approach was taken [48]:

(12) $${\mathrm{CR}}_{i}=\frac{1/{\mathrm{DE}}_{i}}{\sum_{j=1}^{z}\left(1/{\mathrm{DE}}_{j}\right)}\times {\mathrm{DE}}_{0}\times 100\%,$$

where CR*_i_* represents the contribution rate of the *i*th variable, DE*_j_* represents the proportion of the deviance explained by the GAM dropping the *j*th variable, and DE_0_ represents the proportion of deviance explained by the GAM using all *z* variables simultaneously (presumed to have highest predictive power).

All calculations were carried out using R (v4.3.1) [49]. The ADD, ADTS, and ADP methods were carried out with the “spphpr” package (v1.1.4) [35], whilst the GAMs were carried out with the “mgcv” package (v1.9-1) [42,50].

## 3. Results

**Figure 1:**
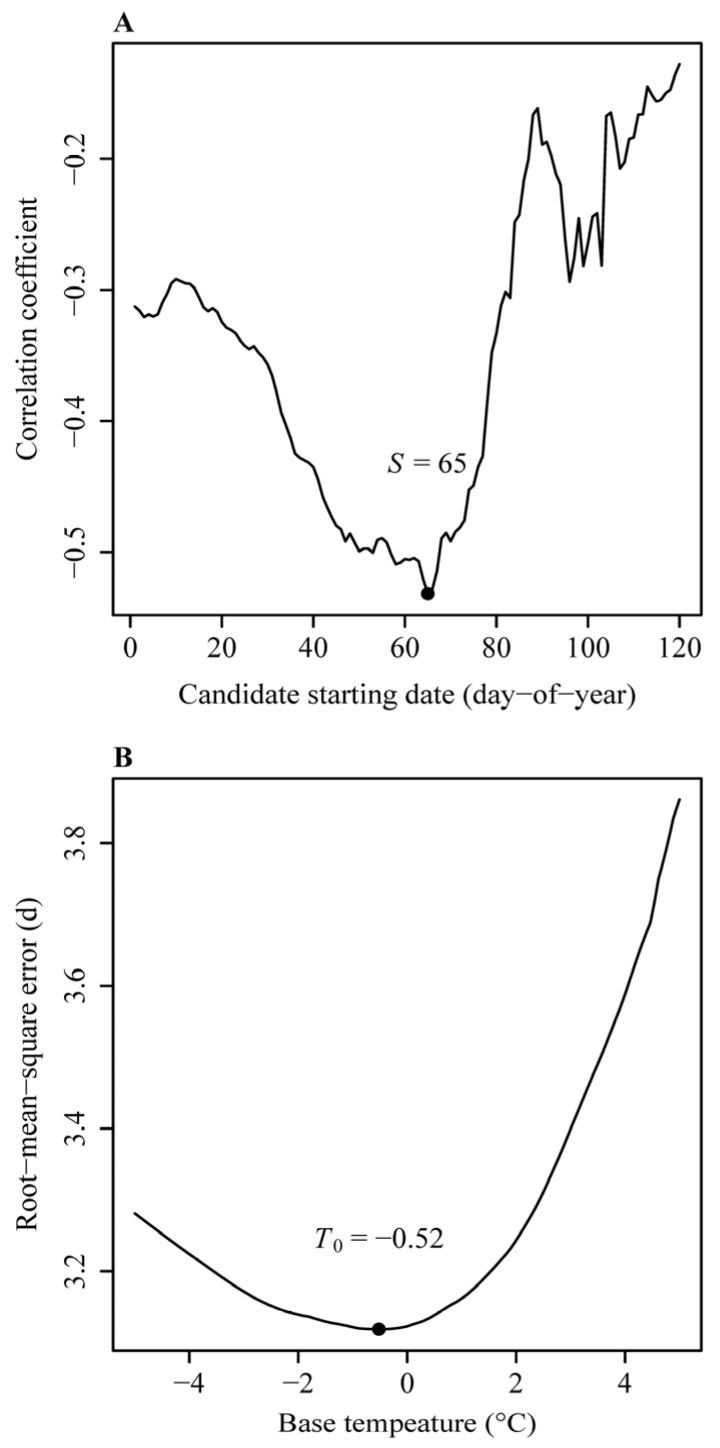
Determination of the starting date and base temperature of the first flowering phenology of *Prunus armeniaca* L. using the accumulated degree-days (ADD) method. (**A**) Correlation coefficients calculated for different starting dates between the first flowering date and the mean value of the daily mean temperatures from the candidate starting date to the observed first flowering date. (**B**) Root mean square error (RMSE) between the observed and predicted occurrence dates when different base temperatures were used in the ADD method. Minimum values (*S* = 65, base temperature = −0.52 °C) are indicated with labeled points on both plots.

**Figure 2:**
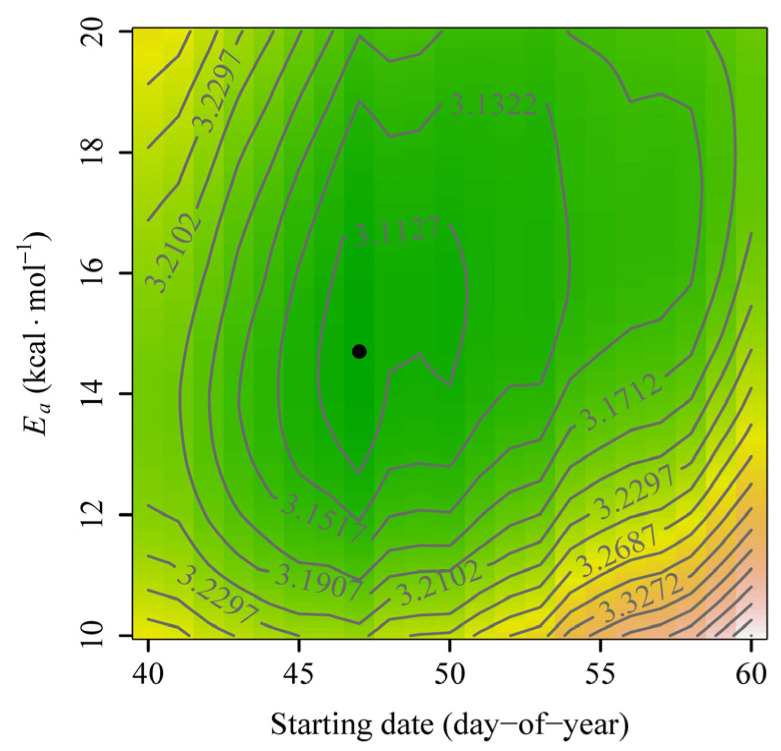
Contour plot of the root mean square errors (RMSEs) resulting from different combinations of starting date and *E_a_* values in the accumulated days transferred to a standardized temperature (ADTS) method. RMSE was minimized when *S* = 47 and *E_a_* = 14.7 kcal∙mol^−1^. The color palettes resembling typical terrain colors were used to represent different RMSE values across different combinations of *S* and *E_a_*.

**Figure 3:**
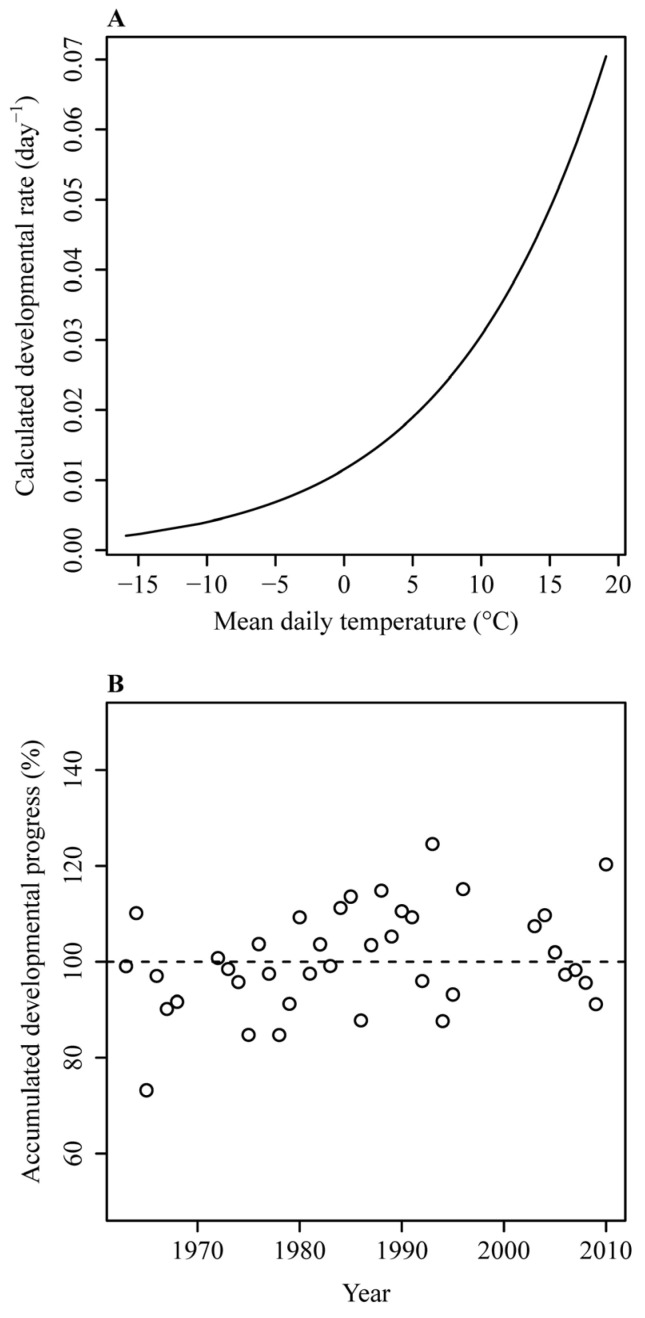
Predicted developmental rates at different temperatures (**A**) and accumulated developmental progresses in different years (**B**) calculated using the accumulated developmental progress (ADP) method based on the Arrhenius equation.

For the accumulated degree-days (ADD) method, the estimated starting date (*S*) was day-of-year (DOY) 65, which was associated with a minimum correlation coefficient (between the occurrence date and the mean of the daily mean temperatures from the candidate starting date to the occurrence date) of −0.53 (Figure 1A). Using this method, the estimated base temperature (i.e., the biological zero degree) was −0.52 °C (Figure 1B), and the root mean square error (RMSE) was 3.1189 days. For the accumulated days transferred to a standardized temperature (ADTS) method, the estimated *S* was DOY 47, the estimated *E_a_* was 14.7 kcal∙mol^−1^, and the RMSE was 3.0932 days (Figure 2). For the accumulated developmental progress (ADP) method, the estimated *S* was DOY 47, the estimated values of *B* and *E_a_* were −4.38 and 15.04 kcal∙mol^−1^, respectively, and the RMSE was 3.0904 days. The predicted temperature-dependent developmental rate curve in the form of the Arrhenius equation (i.e., Equation (4)) based on the ADP method, and the deviation of the calculated developmental progress from the theoretical prediction of 100% across different phenological years, are shown in Figure 3. Among the three methods, the ADP method had the lowest RMSE value.

**Figure 4:**
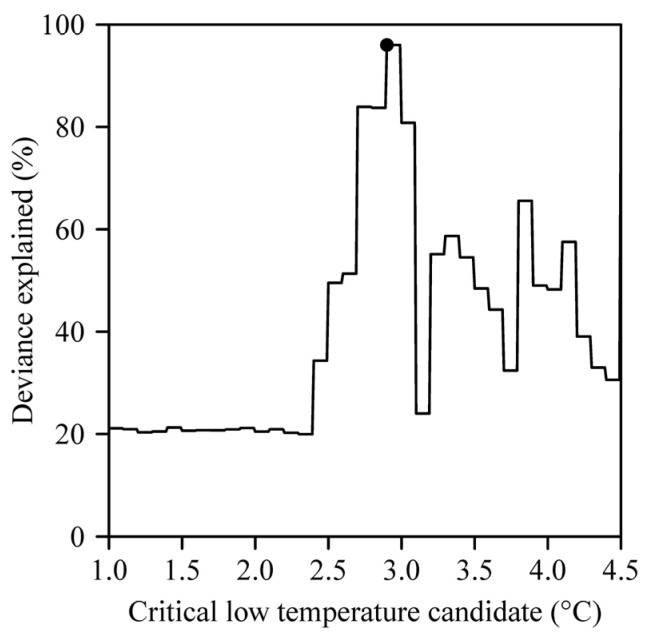
Effects of different candidate low critical temperature values on the deviance explained by the generalized additive model fit. The target critical temperature that accounts for the largest degree of deviance is indicated with a point on the plot.

**Figure 5:**
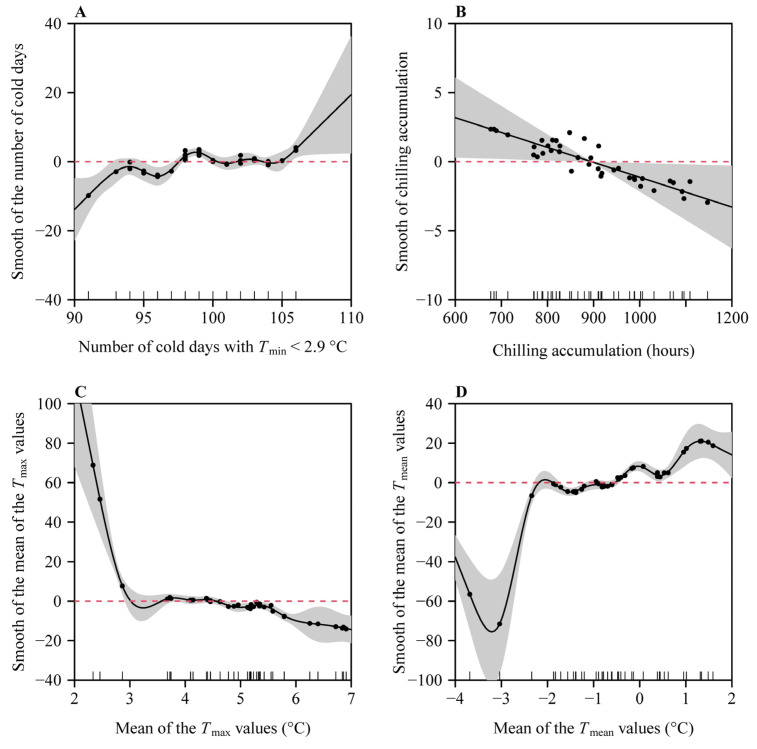
Generalized additive model fits to (**A**) the number of days with daily minimum temperature ≤ 2.9 °C during the period from 1 November of the preceding year to the starting date; (**B**) the number of chilling hours from 1 November of the preceding year to the starting date; (**C**) the mean value of the daily maximum temperatures (*T*_max_) from 1 November of the preceding year to the starting date; (**D**) the mean value of the daily mean temperatures (*T*_mean_) from 1 November of the preceding year to the starting date. In each panel, the dots represent the partial residuals, and the red dashed horizontal line indicates the zero partial residual level.

**Figure 6:**
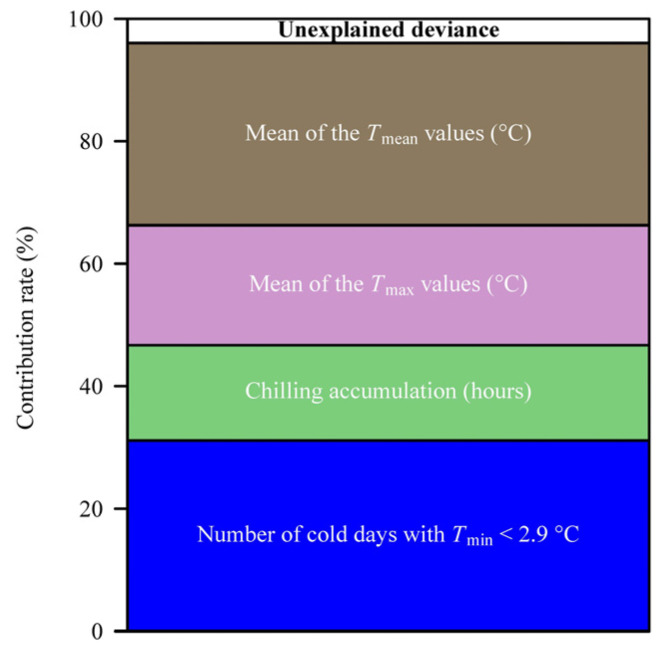
Contribution rates (%) of the four explanatory variables to the deviance explained by the best GAM, as well as the remaining unexplained deviance.

When the critical low temperature was equal to 2.9 °C, the percentage of deviance explained by the generalized additive model (GAM) reached the maximum value of 96% (Figure 4). Analyses using GAMs showed that the number of days with a daily minimum temperature ≤ 2.9 °C (*x*_1_), the number of chilling hours (*x*_2_), the mean value of the daily maximum temperatures (*x*_4_), and the mean value of the daily mean temperatures (*x*_5_) significantly influenced the residuals between the observed occurrence dates and those predicted by the ADP method (Table 1; Figure 5). As the number of days with a daily minimum temperature ≤ 2.9 °C and the mean value of the daily mean temperatures increased, the magnitude of the residuals overall increased; conversely, as the fall and winter chilling hours and the mean value of the daily maximum temperatures increased, the magnitude of the residuals overall decreased (Figure 5). Figure 6 shows the contribution rates of these four variables to deviance explained by the best GAM.

**Figure 7:**
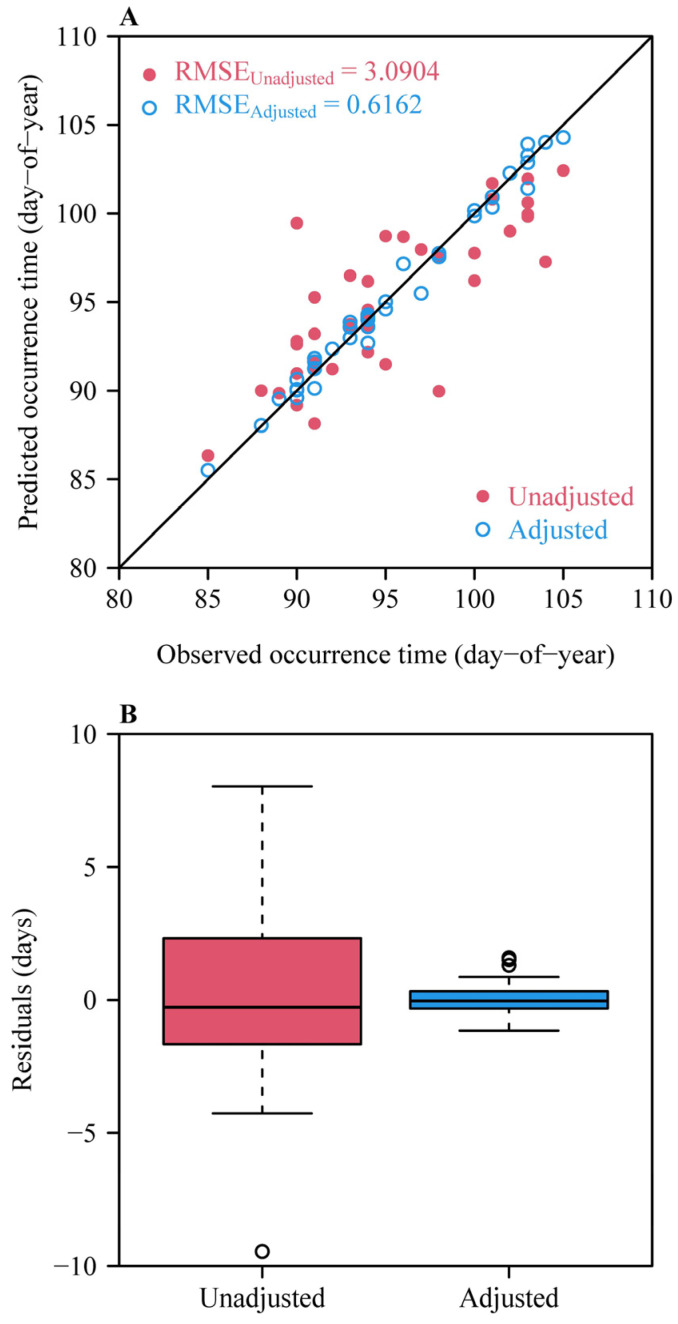
Effects of the four fall and winter temperature (FWT) measures on the predicted residuals when the upper threshold temperature in the Chilling Hours model is equal to 7.2 °C. (**A**) Comparison between the observed and predicted occurrence dates before and after considering the effects of the four FWT measures on the residuals. Here, the red closed circles represent the predicted occurrence dates when not considering the FWT measures, and the blue open circles represent the predicted occurrence dates when considering the effects of the FWT measures. (**B**) Comparison of the predicted residuals without (unadjusted) and with (adjusted) considering the effects of winter temperature measures in the best GAM.

After fitting GAMs considering the influences of the above four fall and winter temperature measures on the residuals between the observed occurrence dates and those predicted by the ADP method, the residuals were further decreased and the RMSE value decreased from 3.0904 to 0.6162 (Figure 7).

## 4. Discussion

Prior work recommended using the logistic equation to describe the effect of spring temperatures on the occurrence date of spring phenological events and a triangular or a trapezoidal curve to describe the effect of fall and winter temperatures (FWTs) on the rate of chilling [14,16,30]. However, as we noted above, the temperature-dependent developmental rate of plants could be depicted well by an exponential equation over low and mid-temperature ranges, especially the range of daily mean temperatures in early spring [21]. It also appears feasible to use a three-parameter logistic equation to describe the effect of early spring temperatures on the developmental rate at temperatures below those at which the developmental rate reaches its maximum [30]. Thus, in the Discussion section below, we compare the performance of the logistic equation (representing a typical sigmoidal curve) and Logan equation (representing a typical left-skewed bell-shaped curve) [12,28] with the Arrhenius equation (a typical exponential curve) in phenological prediction based on the ADP method. Compared with the developmental rate model that has been validated by a large amount of temperature-dependent experiments on arthropods and plants [20,21,25,26,27], the temperature-dependent chilling rate model has seldom been supported in experiments because of the difficulty in determining when chilling accumulation has completed, with the exception of several species-specific cases [16,51]. It is apparent that determining whether chilling accumulation has completed requires carrying out an additional temperature-dependent development experiment, which for tree physiology is usually impractical or too expensive, as doing so requires using several greenhouses for growing adult trees at different controlled constant temperatures. After all, branches or seedlings tend to have different physiological and phenological responses to temperatures from those exhibited by adult trees. In addition, in our analysis of the residuals between the observed occurrence dates and those predicted by the ADP method, we used the Chilling Hours model [45,46] to quantify the fall and winter chilling accumulation. In this procedure we directly defined the upper threshold temperature as 7.2 °C, but this was based on an empirical value from other species [45], leading to uncertainty. One wonders whether it is feasible to replace this estimate with another value, such as 4 °C, for example, which was frequently used in prior studies. Emerging evidence reveals dynamic overlaps between chilling fulfillment and heat accumulation during dormancy release [19], challenging the classical paradigm of strictly sequential chilling–forcing phases [15,16]. This conceptual shift advances our understanding of phenological plasticity, particularly in species occupying transitional climate zones. In this section, we will focus on discussion of the above questions.

### 4.1. Two Other Nonlinear Equations for Describing Temperature-Dependent Developmental Rate

The Logan equation [28] is well-established as a model to describe the effect of temperature on the developmental rate of arthropods, and its form is as follows:

(13) $$r=\psi \left[\exp\left(\rho T\right)-\exp\left(\rho T_{u}-\frac{T_{u}-T}{z}\right)\right],$$

where *ψ*, *ρ*, *T_u_*, and *z* are parameters to be estimated.

The three-parameter logistic equation [29,30] is a typical sigmoid function, whose form is as follows:

(14) $$r=\frac{K}{1+\left(\frac{K}{K_{0}}-1\right)\times \exp\left(-bT\right)},$$

where *K* is the asymptotic maximum developmental rate, *K*_0_ is the theoretical initial developmental rate, and *b* is the rate of increase; these three parameters are constants to be estimated.

We found that using the ADP method with the logistic equation resulted in the lowest RMSE value (3.0881 days) and using the Logan equation resulted in the highest RMSE value (3.090418) among the three nonlinear equations, i.e., the Arrhenius equation, the Logan equation, and the logistic equation (Table 2). However, although the lowest RMSE was achieved by the ADP method using the logistic equation, the response curve of the developmental rate versus temperature predicted using this equation is not correct over the whole thermal range possibly encountered in nature, as the temperature-dependent developmental rate is expected to be a left-skewed bell-shaped curve overall [23,24,25,26,27]. Fortunately, there were small differences in the RMSE values among the ADD method, the ADTS method, and the three types of the ADP method.

### 4.2. Influence of the Upper Threshold Temperature in the Chilling Hours Model on Goodness of Fit

**Figure 8:**
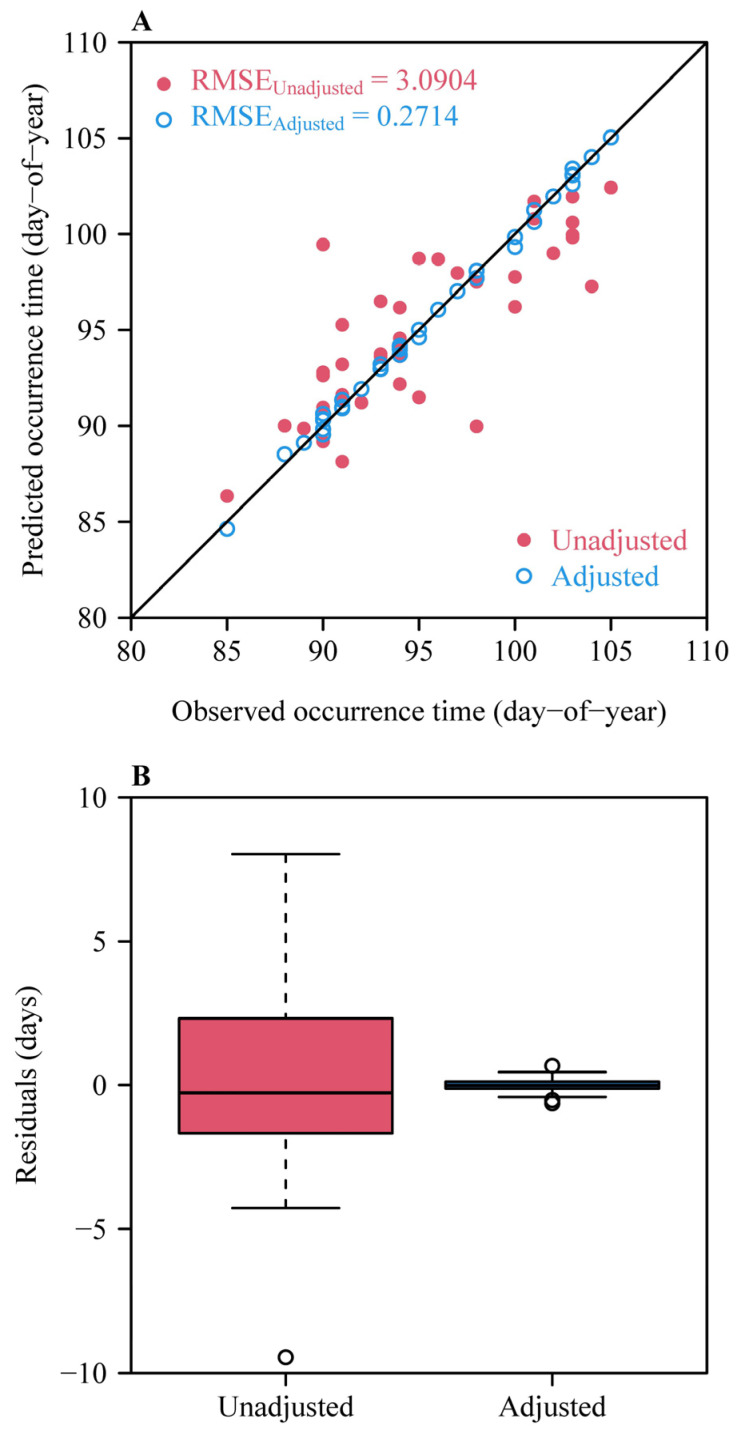
Effects of the four fall and winter temperature (FWT) measures on the predicted residuals when the upper threshold temperature in the Chilling Hours model is equal to 4 °C. (**A**) Comparison between the observed and predicted occurrence dates before and after considering the effects of the four FWT measures on the residuals. Here, the red closed circles represent the predicted occurrence dates when not considering the FWT measures, and the blue open circles represent the predicted occurrence dates when considering the effects of the FWT measures. (**B**) Comparison of the predicted residuals without (unadjusted) and with (adjusted) considering the effects of winter temperature measures in the best GAM.

**Figure 9:**
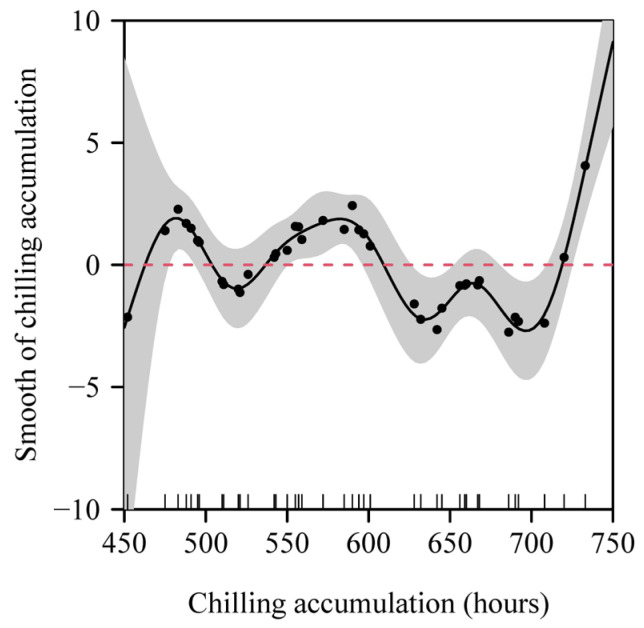
Partial residuals and smooth of the number of chilling hours from 1 November of the preceding year to the starting date (with 4 °C as a candidate upper threshold temperature in the Chilling Hours model) in the generalized additive model. Here, the dots represent the partial residuals, and the red dashed horizontal line indicates the zero partial residual level.

In Equation (10), 7.2 °C was set as the upper threshold temperature for counting one chilling hour in the Chilling Hours model [45,46]. When the upper threshold temperature was set to 4 °C, the percentage of deviance explained increased to 99.2% and the RMSE between the observed and predicted residuals was equal to 0.2714 days (Figure 8). However, the partial residual plot of the candidate model shows the probable overfitting of the data in this case, given that the nonlinear undulant effect of the chilling accumulation on the response variable cannot be accounted for (Figure 9). Other numerical values between 0 °C and 7.2 °C could be considered as the potential upper threshold temperature, but we did not test the influence of these values on the prediction error given the lack of knowledge on what values to test objectively. In the present study, we only compared 7.2 °C with 4 °C (below which there is frost on the grass), because the two temperatures have been frequently used in prior studies [45,46]. In addition, 4 °C was found to be valid for use in calculating the chilling accumulation needed for seed germination [21]. Thus, it is more reasonable to use 7.2 °C as the upper threshold temperature for counting chilling hours, at least until future studies investigate this subject further in apricot trees.

### 4.3. Effect of Daily Maximum Temperatures on Occurrence Date

In a prior study of *P. × yedoensis*, only the number of cold days, the mean value of the daily mean temperatures, and the annual minimum temperature were considered to account for the residuals between the observed FFD values and those predicted by the ADP method [11]. Daily maximum temperatures from 1 November to the starting date have been largely neglected in past studies on this subject. The present study shows that adding the mean value of the daily maximum temperatures during the development period to the FFD prediction model greatly improved its goodness of fit and decreased its prediction error (Table 1; Figure 7). Most prior studies considered the fall and winter chilling accumulation to occur ahead of the accumulation of effective spring temperatures, and thus, it is likely to have little effect on spring phenology. However, results of the present study imply that the accumulation of effective temperatures tends to begin earlier, in late autumn and winter, and thus the breaking of eco-dormancy may occur in two phases: one occurs in fall and early winter but stops when daily maximum temperatures become too low, and another starts in late winter or early spring from the starting date. The ADTS and ADP methods both predicted that the starting date of the accumulation of effective temperatures for *P. armeniaca* occurred in late winter (16 February) in the present study. If the starting date was predicted to be earlier (e.g., 1 January), the RMSE would have become larger. Thus, we hypothesized that the accumulation of effective temperatures in fall and winter was probably terminated due to low temperatures in mid-winter but restarted in late winter when daily air temperatures increased, which was referred to as the parallel model in a prior study [16]. This can explain why the mean value of the daily maximum temperatures was found to be a significant predictor, reducing the prediction error for the ADP method herein (Table 1). However, whether the above hypothetical heat accumulation pattern (i.e., the parallel model) is species-specific or universal for spring-flowering plants merits further investigation.

### 4.4. Overlap Between Chilling and Heat Accumulation: Implications for Phenological Modeling

Recent experimental evidence challenges the traditional assumption of strictly sequential chilling and heat requirements in phenological models. In apricot, Delgado et al. [19] demonstrated that once 75% of cultivar-specific chilling requirements (CRs) are fulfilled, simultaneous accumulation of both chill and heat units can significantly advance flowering dates. This suggests a dynamic overlap between dormancy phases, where partial chilling enables earlier responsiveness to forcing temperatures. This is a phenomenon not captured by classic sequential models (e.g., refs. [16,52]). These findings align with [18], who reported a negative correlation between CRs and heat requirements (HRs) across apricot cultivars, implying that genotypes with higher CRs may require less heat accumulation for flowering, likely due to earlier fulfillment of endo-dormancy release thresholds.

This overlap aligns with the framework proposed by [15], who emphasized that endo-dormancy break timing, denoted as a critical transition point, determines the onset of eco-dormancy and subsequent heat-driven budburst. Their work highlights that models calibrated solely on budbreak dates may fail to account for shifts in endo-dormancy dynamics under warming winters, particularly at species’ equatorial range limits. For instance, insufficient chilling could delay or prevent endo-dormancy break, forcing buds to rely on suboptimal heat accumulation periods. The dynamic interplay between partial chilling and heat responsiveness observed by [19] underscores the need to refine process-based models by incorporating phase overlap mechanisms.

The sequential model, while effective for *Fagus sylvatica* L. under historical climates [16], assumes strict separation between chilling and heat accumulation phases. However, in warmer climates where chilling thresholds are marginally met, such rigidity may lead to systematic prediction errors. Chuine et al. [15] further demonstrated that models neglecting endo-dormancy break dates produce divergent projections in long-term climate scenarios, particularly those extending beyond 2050. Integrating overlapping chilling–heat interactions, as observed in apricot, could improve model realism by allowing partial chilling efficacy to modulate heat responsiveness, akin to the “competence function” proposed by [30] but with temperature-driven thresholds.

These insights call for re-evaluating the assumption of phase separation in phenological models. Future frameworks should test whether dynamic chilling–heat overlaps are species-specific or a generalizable phenomenon, particularly for crops and trees at climatic margins. Additionally, high-resolution measurements of endo-dormancy break dates [15] combined with controlled experiments on chilling–heat synergies [19] will be critical for parameterizing next-generation models capable of capturing nonlinear climate–phenology feedbacks.

### 4.5. Strengths and Limitations of the Methods Presented Here

While the physiological interplay between fall–winter temperatures and flowering phenology in fruit trees is well established (e.g., refs. [7,9]), this study advances the field by developing a quantitative framework (ADP-GAM) that dynamically integrates chilling and heat accumulation processes with temperature thresholds. Unlike traditional sequential models, which rigidly separate chilling fulfillment and heat forcing, our approach reveals how intermittent warm spells during fall-winter, captured through the mean value of daily maximum temperatures, can partially mitigate chilling deficits, a critical adaptation mechanism under erratic winter warming. Furthermore, the model’s cultivar-independent design enhances its applicability in regions lacking detailed phenological registries, enabling farmers to predict flowering dates using locally recorded fall-winter temperatures without prior knowledge of cultivar-specific traits. By identifying actionable thresholds (e.g., ≤2.9 °C cold days) and linking temperature variability to residual flowering shifts, the framework supports climate adaptation strategies, such as selecting genotypes resilient to warmer winters or deploying targeted frost protection measures. Thus, while grounded in known physiology, the model’s predictive flexibility and scalability offer novel tools for precision phenology in horticulture, addressing both scientific and practical gaps in climate-resilient crop management.

However, accumulating evidence underscores the multifaceted climatic drivers of spring phenological shifts. For instance, elevated daytime and nighttime temperatures reduce chilling unit accumulation, thereby delaying spring phenology [53], while late spring frost (LSF) further exacerbates delays in Northern Hemisphere tree phenology by impairing photosynthetic carbon assimilation [54]. Importantly, the methodology proposed here may have limited applicability to species governed primarily by photoperiodic cues. For such plants, daylength must be recognized as the dominant regulator of blooming processes [55]. Future investigations should prioritize integrating photoperiodic controls, thermal thresholds, and frost-mediated carbon constraints within the ADP-GAM framework to disentangle their synergistic or antagonistic effects on phenological trajectories—an advancement critical for refining predictive models under climate change.

## 5. Conclusions

This study demonstrates that the accumulated developmental progress (ADP) method, based on the Arrhenius equation, outperforms traditional models (ADD and ADTS) in predicting apricot first flowering dates (FFDs), achieving the lowest RMSE (3.09 days). By integrating fall and winter temperatures (FWTs) into generalized additive models (GAMs), we identified four critical predictors: the number of cold days (≤2.9 °C), the number of chilling hours, the mean value of daily maximum temperatures, and the mean value of daily mean temperatures from 1 November to the starting date. These predictors collectively explained 96% of the residual variance, emphasizing that chilling accumulation and intermittent warmth jointly regulate dormancy release. These findings challenge the traditional sequential chilling–forcing paradigm and carry significant implications for apricot production under global warming. Rising winter temperatures may reduce chilling fulfillment, necessitating adaptive strategies such as selecting low-chill cultivars or employing artificial chilling to mitigate yield risks. The findings of this study highlight that warmer late winters, by accelerating spring forcing, could advance FFD in apricot. While this research did not explicitly assess frost risks, such phenological shifts may increase susceptibility to late frost events under climate warming scenarios, which is a concern supported by broader agroclimatic studies. To address potential challenges, strategies such as monitoring temperature thresholds and deploying frost-protection measures could be prioritized in regions experiencing rapid warming. Additionally, the observed linkage between chilling requirements and heat responsiveness suggests that breeding programs might benefit from selecting cultivars with reduced chilling demands and enhanced thermal adaptability, aligning with projected climate trajectories.

## Figures and Tables

**Table 1:** Comparison among different candidate generalized additive models explaining residuals of observed versus predicted FFD.

Formula	AIC	Deviance Explained	Non-Significant Items
*y* ~ s(*x*_1_) + s(*x*_2_) + s(*x*_3_) + s(*x*_4_) + s(*x*_5_) + s(*x*_6_)	86.62	99.0%	s(*x*_3_)
*y* ~ + s(*x*_2_) + s(*x*_3_) + s(*x*_4_) + s(*x*_5_) + s(*x*_6_)	203.56	42.1%	All items
*y* ~ s(*x*_1_) + s(*x*_3_) + s(*x*_4_) + s(*x*_5_) + s(*x*_6_)	127.28	96.6%	s(*x*_4_) and s(*x*_6_)
*y* ~ s(*x*_1_) + s(*x*_2_) + s(*x*_4_) + s(*x*_5_) + s(*x*_6_)	167.26	86.5%	s(*x*_6_)
*y* ~ s(*x*_1_) + s(*x*_2_) + s(*x*_3_) + s(*x*_5_) + s(*x*_6_)	192.08	64.5%	s(*x*_5_) and s(*x*_6_)
*y* ~ s(*x*_1_) + s(*x*_2_) + s(*x*_3_) + s(*x*_4_) + s(*x*_6_)	200.05	39.0%	s(*x*_1_), s(*x*_2_), s(*x*_4_), and s(*x*_6_)
*y* ~ s(*x*_1_) + s(*x*_2_) + s(*x*_3_) + s(*x*_4_) + s(*x*_5_)	161.09	89.6%	s(*x*_2_) and s(*x*_3_)
*y* ~ s(*x*_1_) + s(*x*_2_) + s(*x*_4_) + s(*x*_5_)	130.02	96.0%	−
*y* ~ s(*x*_1_) + s(*x*_2_) + s(*x*_5_)	202.52	26.8%	All items
*y* ~ s(*x*_1_) + s(*x*_2_) + s(*x*_4_)	205.80	17.6%	All items

Here, each model used the Gaussian link for the response variable. “Non-significant items” denotes the smooth terms that were not significant at the 0.05 significance level, and “−” means that all items in the corresponding model were significant. Here, *x*_1_ represents the number of days with daily minimum temperature ≤ 2.9 °C, *x*_2_ represents the number of chilling hours, *x*_3_ represents the mean value of the daily minimum temperatures, *x*_4_ represents the mean value of the daily maximum temperatures, *x*_5_ represents the mean value of the daily mean temperatures, and *x*_6_ represents the minimum value of the daily minimum temperatures; all these predictors were calculated from 1 November of the preceding year to the starting date.

**Table 2:** Comparison of parameter estimates of the five methods for predicting the first flowering date of *P. armeniaca*.

Method	Estimate of the Starting Date (DOY)	Estimate(s) of the Model Parameter(s)	RMSE (Days)
ADD	65	*T*_0_ = −0.52 °C	3.1189
ADTS	47	*E_a_* = 14.7 kcal∙mol^−1^	3.0932
ADP–Arrhenius	47	*B* = −4.38	3.090395
		*E_a_* = 15.04 kcal∙mol^−1^	
ADP–Logan	47	*ψ* = 0.01226	3.090418
		*ρ* = 0.1066	
		*T_u_* = 40.63	
		*z* = 5.8179	
ADP–Logistic	47	*K* = 0.1463	3.088134
		*K*_0_ = 0.0114	
		*b* = 0.1148	

Here, ADP–Arrhenius, ADP–Logan, and ADP–Logistic represent the ADP methods based on the Arrhenius, Logan, and logistic equations, i.e., Equations (4), (13) and (14), respectively.

## Data Availability

The first flowering dates of *Prunus armeniaca* L. and corresponding daily air temperature records are available through the “apricotFFD” and “BJDAT” data sets in the R package spphpr (v1.1.4; Shi et al. [35]).

## References

[1] Chmielewski F.M., Schwartz M.D. (2003). Phenology and agriculture. Phenology: An Integrative Environmental Science. Tasks for Vegetation Science.

[2] Schwartz M.D. (2025). Phenology: An Integrative Environmental Science.

[3] Chuine I., Morin X., Bugmann H. (2010). Warming, photoperiods, and tree phenology. Science.

[4] Fu Y.H., Zhao H., Piao S., Peaucelle M., Peng S., Zhou G., Ciais P., Huang M., Menzel A., Peñuelas J. (2015). Declining global warming effects on the phenology of spring leaf unfolding. Nature.

[5] Prevéy J.S., Rixen C., Rüger N., Høye T.T., Bjorkman A.D., Myers-Smith I.H., Elmendorf S.C., Ashton I.W., Cannone N., Chisholm C.L. (2019). Warming shortens flowering seasons of tundra plant communities. Nat. Ecol. Evol..

[6] Visser M.E., Gienapp P. (2019). Evolutionary and demographic consequences of phenological mismatches. Nat. Ecol. Evol..

[7] Heide O.M. (2003). High autumn temperature delays spring bud burst in boreal trees, counterbalancing the effect of climatic warming. Tree Physiol..

[8] Linvill D.E. (1990). Calculating chilling hours and chill units from daily maximum and minimum temperature observations. HortScience.

[9] Luedeling E., Zhang M., Luedeling V., Girvetz E.H. (2009). Sensitivity of winter chill models for fruit and nut trees to climatic changes expected in California’s Central Valley. Agric. Ecosyst. Environ..

[10] Fu Y.H., Campioli M., Deckmyn G., Janssens I.A. (2012). The impact of winter and spring temperatures on temperate tree budburst dates: Results from an experimental climate manipulation. PLoS ONE.

[11] Shi P., Chen Z., Reddy G.V.P., Hui C., Huang J., Xiao M. (2017). Timing of cherry tree blooming: Contrasting effects of rising winter low temperatures and early spring temperatures. Agric. Forest Meteorol..

[12] Shi P., Fan M., Reddy G.V.P. (2017). Comparison of thermal performance equations in describing temperature-dependent developmental rates of insects: (III) Phenological applications. Ann. Entomol. Soc. Am..

[13] Zhang R., Lin J., Wang F., Delpierre N., Kramer K., Hänninen H., Wu J. (2022). Spring phenology in subtropical trees: Developing process-based models on an experimental basis. Agric. Forest Meteorol..

[14] Chuine I. (2000). A unified model for budburst of trees. J. Theor. Biol..

[15] Chuine I., Bonhomme M., Legave J.-M., de Cortázar-Atauri I.G., Charrier G., Lacointe A. (2016). Can phenological models predict tree phenology accurately in the future? The unrevealed hurdle of endodormancy break. Glob. Change Biol..

[16] Kramer K. (1994). Selecting a model to predict the onset of growth of *Fagus sylvatica*. J. Appl. Ecol..

[17] Luedeling E., Kunz A., Blanke M. (2013). Identification of chilling and heat requirements of cherry trees—A statistical approach. Int. J. Biometeorol..

[18] Ruiz D., Campoy J.A., Egea J.A. (2007). Chilling and heat requirements of apricot cultivars for flowering. Environ. Exp. Bot..

[19] Delgado A., Ruiz D., Munoz-Morales A.M., Campoy J.A., Egea J.A. (2025). Analysing variations in flowering time based on the dynamics of chill and heat accumulation during the fulfilment of cultivar-specific chill requirements in apricot. Eur. J. Agron..

[20] Shi P., Reddy G.V.P., Chen L., Ge F. (2016). Comparison of thermal performance equations in describing temperature-dependent developmental rates of insects: (I) empirical models. Ann. Entomol. Soc. Am..

[21] Shi P., Quinn B.K., Zhang Y., Bao X., Lin S. (2019). Comparison of the intrinsic optimum temperatures for seed germination between two bamboo species based on a thermodynamic model. Glob. Ecol. Conser..

[22] Quinn B.K. (2017). A critical review of the use and performance of different function types for modeling temperature-dependent development of arthropod larvae. J. Therm. Biol..

[23] Uvarov B.P. (1931). Insects and climate. Trans. Entomol. Soc. Lond..

[24] Campbell A., Frazer B.D., Gilbert N., Gutierrez A.P., Mackauer M. (1974). Temperature requirements of some aphids and their parasites. J. Appl. Ecol..

[25] Sharpe P.J.H., DeMichele D.W. (1977). Reaction kinetics of poikilotherm development. J. Theor. Biol..

[26] Ratkowsky D.A., Reddy G.V.P. (2017). Empirical model with excellent statistical properties for describing temperature-dependent developmental rates of insects and mites. Ann. Entomol. Soc. Am..

[27] Rebaudo F., Struelens Q., Dangles O. (2017). Modelling temperature-dependent development rate and phenology in arthropods: The devRate package for R. Methods Ecol. Evol..

[28] Logan J.A., Wollkind D.J., Hoyt S.C., Tanigoshi L.K. (1976). An analytic model for description of temperature dependent rate phenomena in arthropods. Environ. Entomol..

[29] Davidson J. (1944). On the relationship between temperature and rate of development of insects at constant temperatures. J. Anim. Ecol..

[30] Hänninen H. (1990). Modelling bud dormancy release in trees from cool and temperate regions. Acta For. Fenn..

[31] Wagner T.L., Wu H.-I., Sharpe P.J.H., Schoolfield R.M., Coulson R.N. (1984). Modelling insect development rates: A literature review and application of a biophysical model. Ann. Entomol. Soc. Am..

[32] Ring D.R., Harris M.K. (1983). Predicting pecan nut casebearer (Lepidoptera: Pyralidae) activity at College Station, Texas. Environ. Entomol..

[33] Aono Y. (1993). Climatological studies on blooming of cherry tree (*Prunus yedoensis*) by means of DTS method. Bull. Univ. Osaka Pref. Ser. B Agric. Life Sci..

[34] Guo L., Xu J., Dai J., Cheng J., Luedeling E. (2015). Statistical identification of chilling and heat requirements for apricot flower buds in Beijing, China. Sci. Horticul..

[35] Shi P., Chen Z., Quinn B.K. (2025). spphpr: Spring Phenological Prediction.

[36] Ring D.R. (1981). Predicting Biological Events in the Life History of the Pecan Nut Casebearer Using a Degree Day Model. Ph.D. Thesis.

[37] Konno T., Sugihara S. (1986). Temperature index for characterizing biological activity in soil and its application to decomposition of soil organic matter. Bull. Natl. Inst. Agro-Environ. Sci..

[38] Ungerer M.J., Ayres M.P., Lombardero M.J. (1999). Climate and the northern distribution limits of *Dendroctonus frontalis* Zimmermann (Coleoptera: Scolytidae). J. Biogeogr..

[39] Nelder J.A., Mead R. (1965). A simplex method for function minimization. Comput. J..

[40] Hastie T.J., Tibshirani R.J. (1986). Generalized additive models (with discussion). Stat. Sci..

[41] Hastie T.J., Tibshirani R.J. (1990). Generalized Additive Models.

[42] Wood S.N. (2017). Generalized Additive Models: An Introduction with R.

[43] Akaike H., Parzen E., Tanabe K., Kitagawa G. (1998). Information theory and an extension of the maximum likelihood principle. Selected Papers of Hirotugu Akaike.

[44] Spiess A.N., Neumeyer N. (2010). An evaluation of *R*^2^ as an inadequate measure for nonlinear models in pharmacological and biochemical research: A Monte Carlo approach. BMC Pharmacol..

[45] Chandler W.H. (1942). Deciduous Orchards.

[46] Luedeling E., Brown P.H. (2011). A global analysis of the comparability of winter chill models for fruit and nut trees. Int. J. Biometeorol..

[47] Zohner C.M., Mo L., Sebald V., Renner S.S. (2020). Leaf-out in northern ecotypes of wide-ranging trees requires less spring warming, enhancing the risk of spring frost damage at cold limits. Glob. Ecol. Biogeogr..

[48] Yang Y., Ratkowsky D.A., Yang J., Shi P. (2023). Effects of plant coverage on the abundance of adult mosquitos at an urban park. Plants.

[49] R Core Team (2023). R: A Language and Environment for Statistical Computing.

[50] Wood S.N. (2011). Fast stable restricted maximum likelihood and marginal likelihood estimation of semiparametric generalized linear models. J. R. Statist. Soc. B.

[51] Zhang R., Lin J., Wang F., Shen S., Wang X., Rao Y., Wu J., Hänninen H. (2021). The chilling requirement of subtropical trees is fulfilled by high temperatures: A generalized hypothesis for tree endodormancy release and a method for testing it. Agric. Forest Meteorol..

[52] Sarvas R. (1974). Investigations on the annual cycle of development of forest trees. II. Autumn dormancy and winter dormancy. Commun. Inst. Fiorestalis Fenn..

[53] Wang J., Xi Z., He X., Chen S., Rossi S., Smith N.G., Liu J., Chen L. (2021). Contrasting temporal variations in responses of leaf unfolding to daytime and nighttime warming. Glob. Change Biol..

[54] Wang J., Hua H., Guo J., Huang X., Zhang X., Yang Y., Wang D., Guo X., Zhang R., Smith N.G. (2025). Late spring frost delays tree spring phenology by reducing photosynthetic productivity. Nat. Clim. Change.

[55] Meng L., Zhou Y., Gu L., Richardson A.D., Peñuelas J., Fu Y., Wang Y., Asrar G.R., de Boeck H.J., Mao J. (2021). Photoperiod decelerates the advance of spring phenology of six deciduous tree species under climate warming. Glob. Change Biol..

